# Extracellular vesicle‐enclosed miR‐486‐5p mediates wound healing with adipose‐derived stem cells by promoting angiogenesis

**DOI:** 10.1111/jcmm.15387

**Published:** 2020-07-14

**Authors:** Yingjie Lu, Huicai Wen, Jinjun Huang, Peng Liao, Huaiwei Liao, Jun Tu, Yuanlin Zeng

**Affiliations:** ^1^ Department of Plastic Surgery The First Affiliated Hospital of Nanchang University Nanchang China; ^2^ Department of Integrated Chinese and Western Medicine The First Affiliated Hospital of Nanchang University Nanchang China; ^3^ Department of Burn Surgery The First Affiliated Hospital of Nanchang University Nanchang China

**Keywords:** adipose‐derived stem cells, angiogenesis, cutaneous wound, extracellular vesicles, microRNA‐486‐5p

## Abstract

Adipose‐derived stem cells (ASC) are said to have a pivotal role in wound healing. Specifically, ASC‐secreted extracellular vesicles (EV) carry diverse cargos such as microRNAs (miRNAs) to participate in the ASC‐based therapies. Considering its effects, we aimed to investigate the role of ASC‐EVs in the cutaneous wound healing accompanied with the study on the specific cargo‐medicated effects on wound healing. Two full‐thickness excisional skin wounds were created on mouse dorsum, and wound healing was recorded at the indicated time points followed by histological analysis and immunofluorescence staining for CD31 and α‐SMA. Human skin fibroblasts (HSFs) and human microvascular endothelial cells (HMECs) were co‐cultured with EVs isolated from ASC (ASC‐EVs), respectively, followed by the evaluation of their viability and mobility using CCK‐8, scratch test and transwell migration assays. Matrigel‐based angiogenesis assays were performed to evaluate vessel‐like tube formation by HMECs in vitro. ASC‐EVs accelerated the healing of full‐thickness skin wounds, increased re‐epithelialization and reduced scar thickness whilst enhanced collagen synthesis and angiogenesis in murine models. However, miR‐486‐5p antagomir abrogated the ASC‐EVs‐induced effects. Intriguingly, miR‐486‐5p was found to be highly enriched in ASC‐EVs, exhibiting an increase in viability and mobility of HSFs and HMECs and enhanced the angiogenic activities of HMECs. Notably, we also demonstrated that ASC‐EVs‐secreted miR‐486‐5p achieved the aforesaid effects through its target gene Sp5. Hence, our results suggest that miR‐486‐5p released by ASC‐EVs could be a critical mediator to develop an ASC‐based therapeutic strategy for wound healing.

## 1 INTRODUCTION

Being characterized as a complicatedly programmed process, cutaneous wound healing involves a wide range of molecular movements including cellular proliferation and migration.^1^ Besides, cutaneous wound healing is also a chronic process in clinical studies and mainly caused by poor angiogenesis.^2^ However, excessive inflammation is considered a major obstacle in cutaneous wound healing.^3^ Long‐lasting cutaneous wound healing has been reported to cause a huge financial burden to the patients whilst the healing results remain unsatisfactory.^4^ To overcome the barrier of cutaneous wound healing, improvement of the new vessel growth, namely, angiogenesis, and stimulation of regenerative tissues are endorsed for subsequent research.^5^ Adipose‐derived stem cells (ASC) have been reported to promote angiogenesis through cell migration and tissue repair.^6^ Because ASCs secrete multifunctional extracellular vesicles (EVs), and has been speculated as multi‐potent tools regarding regenerative medicine and cell therapies.^7^ Nevertheless, the ASC‐released EVs (referred to ASC‐EVs) hold the potential for promising therapeutic effects on angiogenesis and tissue regeneration via microRNAs (miRNAs).^8^ Of note, the miRNAs from ASC‐EVs are implicated in the wound repair promotion.^9^

Besides, miRNAs are a group of non‐coding RNAs, which could alter the expression of genes.^10^ Whilst miR‐486‐5p has been reported in ASC‐EVs through screening of ASC as well as human bone marrow mesenchymal stem cells.^11^ However, the functional roles of miR‐486‐5p in cutaneous wound healing have not been reported yet. Therefore, the ASC‐EVs‐derived miR‐486‐5p requires further investigation to completely understand its mechanism. Intriguingly, our data from the TargetScan database revealed that Sp5 was specifically bound by miR‐486‐5p. Previously, Sp5 has been reported to suppress the growth of vascular endothelial cells in prostate cancer.^12^ Noticeably, Sp5 has also been reported to regulate the Wnt/β‐catenin signalling‐induced cyclin D (CCND) expression in central nervous system^13^ whilst depleted CCND2 expression impairs the endothelial cell repair in atherosclerosis.^14^ Based on the above, we assumed that ASC‐EVs‐secreted miR‐486‐5p mediates the process of cutaneous wound healing by direct binding to the Sp5 gene.

## 2 METHODS AND MATERIALS

### 2.1 Ethics statement

This study was performed with permission from the Ethics Committee of the First Affiliated Hospital of Nanchang University and conducted according to the *Declaration of Helsinki* principles. The signed informed consent was obtained from every participant. All procedures were approved by the Animal Research Committee of the First Affiliated Hospital of Nanchang University.

### 2.2 Isolation of ASC

The human subcutaneous adipose tissue samples were collected from 11 female donors (aged 32‐67 with a mean age of 51 years old) received plastic surgery in the First Affiliated Hospital of Nanchang University and treated according to the previously established method within 24 hours.^15^ Whilst the adipose tissues were isolated from the abdomen after resection. Briefly, the adipose tissues were minced and detached with 0.1% collagenase A (Roche Diagnostics, Mannheim, Germany) in phosphate‐buffered saline (PBS) containing 1% bovine serum albumin (BSA) (Roche Diagnostics) for 45 minutes at 37°C under continuous shaking. Cells subjected to Ficoll density gradient centrifugation (Lymphoprep, Axis‐Shield, Oslo, Norway) were seeded in plates with a density of 1 × 10^5^ cells/cm^2^. After 4 days, non‐adherent cells were removed and the medium was replaced. ASC were cultured in the alpha minimum essential medium (α‐MEM) (Lonza Life Science, Breda, the Netherlands) containing 100 U/mL penicillin, 100 μg/mL streptomycin (Gibco BRL, Grand Island, NE, USA), 10% foetal bovine serum (FBS) or platelet lysate (PL), and 10 U/mL heparin (Leo Pharma, Amsterdam, the Netherlands) in 5% CO_2_ at 37°C under humid condition. Thereafter, the MSC's surface markers were detected using flow cytometry. Briefly, the ASC was made into a single cell suspension, washed by calcium‐ and magnesium‐free PBS, and added with 10% normal goat serum to block non‐specific interaction. Meanwhile, cells were incubated with fluorescein isothiocyanate (FITC)‐labelled monoclonal antibodies to CD14, CD19, CD105, CD34, CD44, CD45, CD73, CD90 and HLA‐DR (1:100; BioLegend, San Diego, CA, USA) for 30 minutes. The FITC‐immunoglobulin G (IgG) was set as the isotype control, washed by PBS, resuspended by 10% normal goat serum and analysed by CyAn ADP Analyzer (Beckman Coulter, Brea, CA, USA).

### 2.3 ASC multi‐differentiation

The differentiation of osteoblasts, adipocytes and chondrocytes was performed according to the previously described methods with slight modification.^16^ Following, after the cells were subjected to the Alizarin red S staining, Oil Red O staining and Alcian Blue staining, respectively, and observed under an optical microscopy.

### 2.4 Extraction of EVs

The EVs were extracted from subcultured ASC (approximately 3.2 × 10^7^ cells). The confluent ASCs were cultured in the α‐MEM containing EVs‐depleted FBS and PL for 24‐48 hours. After centrifugation at 70 000 *g* and 4°C overnight, EVs‐depleted FBS and PL were obtained. Then, EVs were centrifuged following the details elaborated in the previous study.^17^ All ultracentrifugation steps were performed at 4°C in a Beckman ultracentrifuge (Optima L‐90K; Beckman Instruments, Inc) with SW‐32Ti rotor. Cells were resuspended by 200 µL PBS and stored at −80°C.

### 2.5 Identification of EVs

The size distribution of ASC‐EVs was measured by nanoparticle tracking analysis (NTA) using the Nanosizer™ instrument (Malvern Instruments, Malvern, UK). The morphological analysis of EVs was conducted using the Hitachi H‐7650 transmission electron microscope (TEM) (Hitachi, Tokyo, Japan). EVs particles were dissolved in radio‐immunoprecipitation assay buffer and quantitated using the Bicinchoninic Acid Kit (Thermo Fisher Scientific, Rockford, IL, USA). EVs marker proteins (Abcam Inc, Cambridge, UK), CD63 (ab216130, 1:1000), tumour susceptibility gene (TSG101) (ab30871, 1:1000) and the negative control (NC) endoplasmic reticulum marker calnexin (ab22595, 1:1000, Abcam Inc) were studied using Western blot analysis.

### 2.6 Establishment of the cutaneous wound mouse model

A total of 25 Kunming male mice (aged 6‐8 weeks; weighed 18‐22 g) were anesthetized by the intraperitoneal injection of 50 mg/kg pentobarbital sodium (Sigma‐Aldrich, St. Louis, MO, USA) before the operation. The mice were shaved, and dorsum was created with two full‐thickness excisional skin wounds (12‐mm‐diameter). All the mice were subcutaneously injected with ASC‐EVs (200 μg dissolved in 100 μL PBS) or an equal volume of PBS around the wounds at 4 injection sites (25 μL per site), whereas the other group of the mice received a concomitant injection of miR‐486‐5p antagomir or antagomir NC. After 8 days of surgical procedure, mice were killed by an overdose of anaesthesia, with the skin specimens harvested. Skin samples were analysed by histopathological methods.

### 2.7 Wound closure rating

The wounds of each mouse were recorded at 0, 2, 5 and 8 days after surgical procedure with a caliper ruler. The area of the wound was assessed using Image‐Pro Plus 6 software (Media Cybernetics, Bethesda, MD, USA). The reduction of wound size was calculated using the equation established before.^18^

### 2.8 Histological analysis and immunofluorescence examinations

The mouse wound samples and the peripheral healthy skin samples were collected for further study. Skin tissues were fixed by 4% paraformaldehyde solution, dehydrated by gradient ethanol, paraffin‐embedded and sectioned into 10‐μm slices. Tissues were stained by haematoxylin and eosin (HE) and photographed by the optical microscope. The percentage of re‐epithelialization was measured using the previous formula.^19^ The type of collagen was determined using Masson's trichrome staining whilst the extent of newly formed capillaries during the wound healing process was evaluated by immunofluorescence staining for CD31. The antibodies against CD31 (ab28364; 1:50; Abcam Inc), α‐SMA (ab5831; 1:1000; Abcam Inc) and the Cy3‐conjugated secondary antibody (ab97075, 1:250; Abcam Inc) were involved in this experiment. The operation details were referred to as the methods described in the previous work.^18^

### 2.9 Cell culture

Human skin fibroblasts (HSFs) (FuHeng Biology, Shanghai, China) and human microvascular endothelial cells (HMECs) (Cell Bank of the Chinese Academy of Sciences, Shanghai, China) were cultured in the same condition as previous work described.^18^

### 2.10 EVs uptake by HSFs and HMECs

To determine the ASC‐EVs uptake by HSFs and HMECs, EVs were labelled with a green fluorescent dye (PKH67; Sigma‐Aldrich) according to the previously described protocol and then incubated with HSFs and HMECs at 37°C for 3 hours. The cells were washed with PBS and fixed in 4% paraformaldehyde for 15 minutes followed by the 4',6‐diamindino‐2‐phenylindole (DAPI) (0.5 µg/mL; Invitrogen, Carlsbad, CA, USA) staining on the nuclei. The fluorescence microscopy was used to detect the green signals in HSFs and HMECs. The recipient cells stimulated with ASC‐EVs for 3 hours were harvested to assess the miRNA transfer from EVs to HSFs and HMECs. The miRNA expression was analysed by reverse transcription–quantitative PCR (RT‐qPCR).

### 2.11 Cell transfection

Adipose‐derived stem cells were transfected with miR‐486‐5p inhibitor and the relevant NC whereas HSFs and HMECs were transfected with pcDNA‐3.1, pcDNA‐Sp5, siRNA targeting CCND2, miR‐486‐5p mimic and the corresponding NC. The miR‐486‐5p inhibitor, miR‐486‐5p mimic plasmids and their NC were synthesized by Invitrogen Inc. The rest plasmids were purchased from GenePharma (Shanghai, China). Cells were transfected following the instructions of Lipofectamine 2000 (Invitrogen). After transfection, cells were further cultured in 5% CO_2_ at 37°C for 6‐8 hours, followed by centrifugation. After that, the original medium was renewed by complete culture medium for further culturing for 24‐48 hours.

### 2.12 Dual‐luciferase reporter gene assay

The artificially synthesized Sp5 3′‐untranslated region (UTR) fragments were introduced into the psiCHECK‐2 vector (Promega Corporation, Madison, WI, USA). The complementary sequence mutation site was designed on the Sp5 wild type (WT), which was inserted into psiCHECK‐2 vector reporter plasmid. The correctly sequenced luciferase reporter plasmid Sp5 3′UTR‐WT (100 ng) and Sp5 3′UTR‐mutant type (MUT) (100 ng) were transfected with miR‐486‐5p mimic and mimic NC (2 nmol/L; Dharmacon Research, Inc, Waltham, MA, USA) into HEK‐293T cell (CRL‐1415; Shanghai Xin Yu Biotech Co., Ltd, Shanghai, China). After 48 hours, cells were collected and lysed. The luciferase activity was detected using a luciferase detection kit (RG005; Beyotime Biotechnology, Shanghai, China) on a Glomax20/20 fluorescence detector (Promega Corporation).

### 2.13 Cell migration assays

Cell migration was studied using the scratch test and Transwell assay. For the scratch test, HMECs and HSFs were seeded in a 12‐well plate with a density of 2 × 10^5^ cells/well and incubated to reach confluence. The monolayer was scratched using a tip and washed by a serum‐free medium to remove detached cells. The HSFs were photographed at 0 and 24 hours whilst the HMECs were photographed at 0 and 12 hours after the operation. The wound healing area was calculated with the previously established formula.^18^

In the Transwell assay, cells were cultured in a medium with 5% FBS and seeded into the apical chamber of transwell 24‐well plates (Corning Incorporated, Corning, NY, USA) with 8 μm pore filters. Meanwhile, the complete medium containing 50 μg/well ASC‐EVs, miR‐486‐5p mimic or inhibitor and 10% FBS was added into the basolateral chamber. After 12 hours, the migrated cells of the surface were stained with 0.5% crystal violet. The level of migration was observed under an optical microscope (Leica DMI6000B; Leica Microsystems, Wetzlar, Germany) with 6 fields of view randomly selected.

### 2.14 Cell counting kit‐8 assay

The cell proliferation was determined using Cell counting kit‐8 (CCK‐8; Dojindo, Kyushu Island, Japan). Briefly, cells were seeded in a 96‐well plate with 5 × 10^3^ cells/well followed by addition of CCK‐8 solution (10 μL) and 100 μL of fresh medium and incubated at 37°C for 1 hour. The following procedures were performed following the methods described in previous work with slight modification.^18^

### 2.15 Matrigel‐based angiogenic assays

The gel was formed by the incubation of Growth Factor Reduced Matrigel (BD Biosciences, New Jersey, NJ, USA) in 96‐well plates at 37°C for 30 minutes. Then, HMECs were seeded in the 96‐well plates contained gel with 2 × 10^4^ cells/well. After incubation at 37°C for 6 hours, Matrigel‐based was observed by an inverted microscope (Leica DMI6000B; Leica Microsystems, Wetzlar, Germany) and Image‐Pro Plus 6 software.

### 2.16 RNA isolation and quantification

Total RNA was extracted from tissues or cells using the Trizol Reagent (Invitrogen). The primers used in this study were listed in Table 1. EVs‐derived miRNA was isolated using the SeraMir Exosome RNA Purification Kit (System Biosciences, Mountain View, CA, USA). The complementary DNA was synthesized following the instructions of the TaqMan microRNA assay kit (Applied Biosystems, Foster City, CA, USA). The RT‐qPCR reaction was performed using FastStart Universal SYBR Green Master Mix (Roche, Indianapolis, IN, USA) with the miRNA‐specific forward primer (Sangon Biotech, Shanghai, China) and the universal reverse primer provided by the TaqMan microRNA assay kit. U6 small nuclear RNA was used to normalize the results.

**TABLE 1:** Primer sequences for RT‐qPCR

	Primer sequences (5′‐3′)
miR‐486‐5p	F: GAATTTGGAGTTTAGTTATAGTTTTTATT
	R: CCCAACACCACACACACCATACTA
U6	F: CTCGCTTCGGCAGCACA
	R: AACGCTTCACGAATTTGCGT

Abbreviations: F, Forward; miR‐486‐5p, microRNA‐486‐5p; R, Reverse.

### 2.17 Western blot analysis

Proteins were separated by sodium dodecyl sulphate‐polyacrylamide gel electrophoresis (SDS‐PAGE) and transferred to polyvinylidene fluoride membranes (Immobilon P, Millipore, Billerica, MA, USA). The membranes were blocked with 5% milk in Tris‐buffered saline containing 0.1% Tween‐20 for 1 hour at room temperature and incubated with primary antibodies (Abcam Inc) to Sp5 (ab103835, 1:100) and CCND2 (ab230883, 1:1000) at 4°C overnight. Then, the membranes were incubated with the horseradish peroxidase‐conjugated secondary anti‐rabbit IgG (#7074, 1:5000; Cell Signaling Technology, Danvers, MA, USA). The immune‐reactive bands were visualized by enhanced chemiluminescence reagent (Thermo Fisher Scientific) and imaged by the ChemiDoc XRS Plus luminescent image analyser (Bio‐Rad, Hercules, CA, USA). The relative protein expression was analysed by Image‐Pro Plus 6.0 software and the expression of target protein was normalized to the band intensity of glyceraldehyde‐3‐phosphate dehydrogenase (GAPDH).

### 2.18 Statistical analysis

All data are shown as means ± standard deviation, representative of three independent experiments. Conforming to the normal distribution and homogeneous variance, unpaired data between the two groups were analysed unpaired *t* test. Means of multiple groups were compared with the one‐way analysis of variance (ANOVA) with Tukey's test. Data at different time points were compared using repeated‐measures ANOVA followed by Bonferroni's test. Statistical analysis was conducted using SPSS 21.0 version (IBM, Armonk, NY, USA), and *P* < 0.05 was considered statistically significant.

## 3 RESULTS

### 3.1 Characterization of ASC‐derived EVs

**FIGURE 1:**
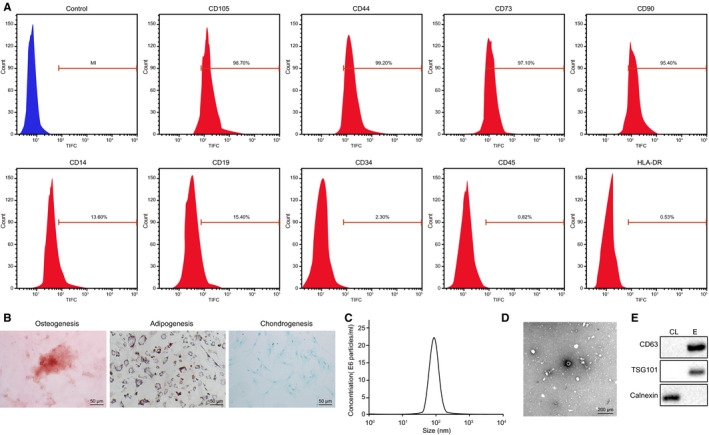
Isolation and identification of ASC‐EVs. A, ASC surface markers determined using flow cytometry. B, Alizarin red staining on cells to detect osteogenesis differentiation (×200); Oil red O staining on cells to study adipogenesis differentiation (×200); Alcian Blue staining on cells to observe chondrogenesis differentiation (×200) (from left to right). C, The size distribution of ASC‐EVs measured by NTA. D, Morphological analysis of EVs dependent on TEM (×50 000). E, EVs specific surface markers protein detected by Western blot analysis. CL, cell lysate; E, extracellular vesicles

To detect the successful isolation of ASC from human adipose tissues, flow cytometry, NTA and TEM analysis were performed. The identification of EVs surface markers by flow cytometry revealed the positive expression of CD105 (98.7%), CD44 (99.2%), CD73 (97.1%) and CD90 (95.4%) accompanied with the negative expression of CD14 (13.6%), CD19 (15.4%), CD34 (2.3%), CD45 (0.82%) and HLA‐DR (0.53%) (Figure 1). Additionally, the isolated cells exhibited the osteogenesis, adipogenesis and chondrogenesis differentiation properties (Figure 1B). Collectively, these results suggested that the isolated ASCs were equipped with MSCs properties.

Furthermore, NTA measurement demonstrated that the diameter of ASC‐derived EVs was ranged from 30 to 100 nm. The results of TEM presented a cyathiform or spherical form of ASC‐EVs. Moreover, EVs surface markers were observed by Western blot analysis, which revealed that the expression of CD63 and TSG101 was increased in EVs in contrast to the cell lysate; however, the expression of calnexin was reduced (Figure 1C‐E). All these results manifested that the ASC‐derived EVs were successfully isolated from human adipose tissues.

### 3.2 ASC‐EVs promote cutaneous wound healing

**FIGURE 2:**
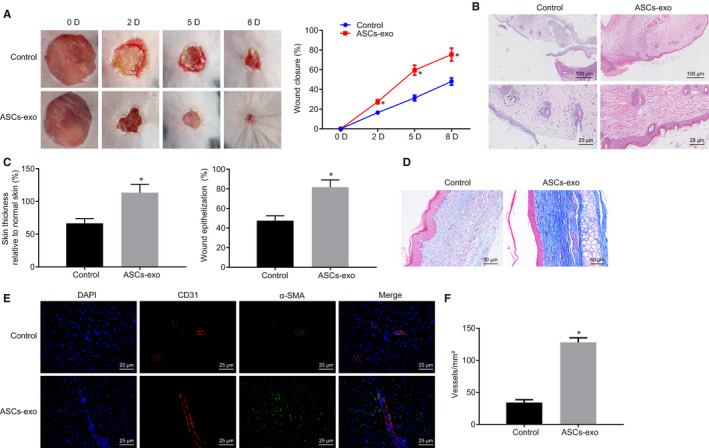
Promoting effect of ASC‐EVs injection on mouse wound healing. A, The representative wound pictures and wound healing rate recorded on the 2nd, 5th and 8th day after injection of ASC‐EVs or PBS (5 millimetres). B, The wound on the 8th day after injection of ASC‐EVs or PBS detected by HE staining (up: 100 µm; down: ×400). C, Scar thickness and wound re‐epithelization measurement. D, Masson's trichrome staining on the wound injected with ASC‐EVs or PBS (×200). E, Immunofluorescence staining on CD31 and a‐SMA of the wound injected with ASC‐EVs or PBS (×400). F, The amount of re‐epithelialization in Figure E quantification. *P < 0.05 compared with the control group. The measurement data were expressed as mean ± standard deviation. The analysis between the two groups was performed by unpaired *t* test. Data amongst multiple groups at different time points were analysed by repeated‐measures ANOVA followed by Bonferroni's test for n = 5

To evaluate the effect of ASC‐EVs on cutaneous wound healing, the full‐thickness‐excised skin wound was created on the mouse dorsum. Then, the subcutaneous injection of ASC‐EVs or PBS with the same volume (as control) was performed near the wound. The process of wound healing with different treatment was recorded and observed through photographs of wound taken at various time duration followed by calculation of wound size reduction. Our results showed that the skin wound was healed more quickly when treated with an injection of ASC‐EVs compared with the injection of PBS. Similar results were shown in the calculation of wound size reduction (Figure 2A). HE staining results exhibited that wound tissues at day 8 exhibited close resemblance with the appearance of new skin, regenerative hair follicle and adipocytes after the wound received ASC‐EVs injection whereas the injection of PBS did not induce the above results (Figure 2B). Additionally, the injection of ASC‐EVs also promoted wound re‐epithelization and hindered the formation of scars (Figure 2C). The collagen type was assessed by Masson's trichrome staining, which demonstrated that in comparison with the control, more collagenous fibres were curved in the wound injected with ASC‐EVs (Figure 2D). Taken together, these results indicated that the ASC‐EVs injection could accelerate the process of wound healing in mice.

The impact of ASC‐EVs on wound area angiogenesis was further explored. The extent of angiogenesis in wound sites was evaluated by the immunofluorescence staining for CD31 and α‐SMA. The results showed that in contrast to the PBS treatment, more angiogenesis (defined by the amount of CD31‐ and α‐SMA‐stained positive cells) appeared in the wound with ASC‐EVs injection (Figure 2E,F). The aforementioned results suggested that ASC‐EVs treatment was able to facilitate the angiogenesis of the mouse wound area.

### 3.3 ASC‐EVs‐secreted miR‐486‐5p accelerates HSFs proliferation and migration

**FIGURE 3:**
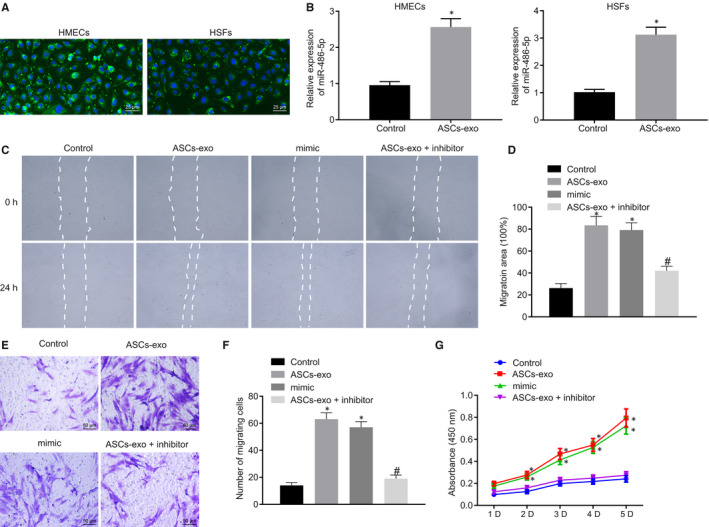
miR‐486‐5p derived from ASC‐EVs accelerates HSFs proliferation and migration. A, ASC‐EVs internalized in HMECs and HSFs observed by fluorescence microscopy. EVs with green fluorescence were observed in recipient cells (×400). B, The expression of miR‐486‐3p in HMECs and HSFs treated with ASC‐EVs or PBS determined using RT‐qPCR after 3 h. C and D, The Migration rate of HSFs with different treatment measured and quantified using the scratch test. E and F, The Migration rate of HSFs with different treatment examined and quantified using Transwell assay (×400). G, Proliferation of HSFs assessed by CCK‐8 assay. *P < 0.05 compared with the control group. ^#^ *P* < 0.05 compared with the ASC‐EVs treatment. The measurement data were described as means ± standard deviation. Data between the two groups were analysed by unpaired *t* test whilst data amongst multiple groups were analysed by one‐way ANOVA followed by Tukey's test. Data at different time points were compared using repeated‐measures ANOVA with Bonferroni's test

To understand the role of ASC‐EVs‐secreted miR‐486‐5p transfer in cells, the ASC‐EVs were labelled by the green fluorescent dye (PKH67) and incubated with HSFs and HMECs. After 3 hours, the fluorescence microscopy analysis showed green fluorescence amongst HSFs and HMECs (Figure 3A) indicating that ASC‐EVs were endocytosed by cells. Moreover, after the ASC‐EVs were incubated with HMECs and HSFs, miRNA was extracted and the expression of miR‐486‐5p was determined using RT‐qPCR. As shown in Figure 3B, miR‐486‐5p expression in HSFs and HMECs treated with ASC‐EVs was remarkably increased relative to the cells treated with PBS. Our data provided evidence that ASC‐EVs shuttled miR‐486‐5p into HMECs and HSFs.

The role of ASC‐EVs in HSFs was further investigated. HSFs were transfected with miR‐486‐5p mimic or treated with ASC‐EVs. Compared with the treatment of PBS, mimic NC and inhibitor NC showed increased migration rate in HSFs treated with ASC‐EVs and the HSFs transfected with miR‐486‐5p mimic. The pro‐migratory ability of ASC‐EVs was further verified by the results of the transwell assay and scratch test. The migration rate in the treatment of ASC‐EVs with miR‐486‐5p inhibitor was significantly declined than the sole treatment of ASC‐EVs (Figure 3C‐F). Meanwhile, the vitality of HSFs was detected and our data exhibited that the HSFs were more active after treatment of ASC‐EVs and the transfection of miR‐486‐5p mimic. However, the HSFs treated with ASC‐EVs with miR‐486‐5p inhibitor were less active than the HSFs with the sole treatment of ASC‐EVs (Figure 3G). The above‐mentioned results indicated that ASC‐EVs‐secreted miR‐486‐5p accelerated the proliferation and migration of HSFs.

### 3.4 ASC‐EVs‐secreted miR‐486‐5p stimulates the angiogenesis of HMECs

**FIGURE 4:**
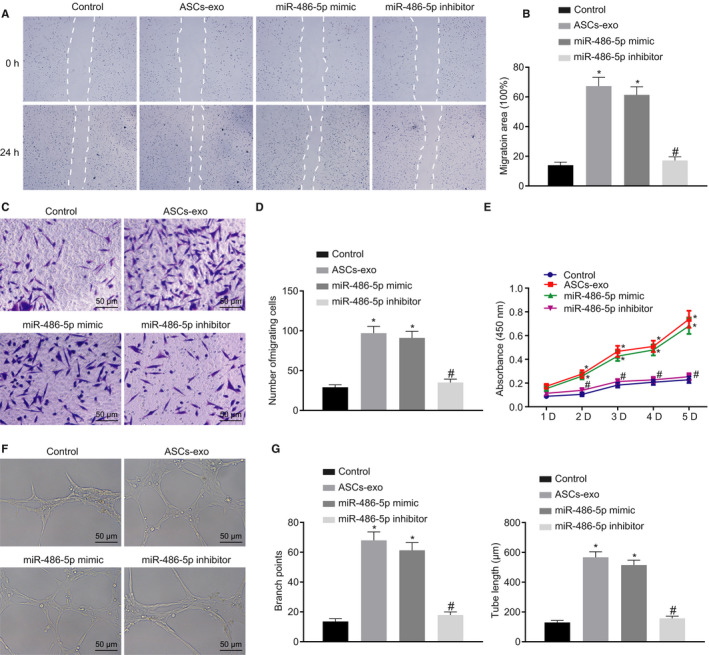
miR‐486‐5p delivered in ASC‐EVs accelerates angiogenesis in HMECs. A and B, Migration of HMECs detected and quantified using the scratch test. C and D, Migration of HMECs examined and quantified using Transwell assay (×200). E, Cell proliferation monitored using CCK‐8 assay. F, Tube formation ability of HMECs assessed by Matrigel‐based assay (×200). G, Tube length and branch points in Figure F quantification. *P < 0.05 compared with the control group. ^#^ *P* < 0.05 compared with the treatment of ASC‐EVs. The measurement data were described as means ± standard deviation. Data between two groups were analysed by unpaired *t* test, whilst data amongst multiple groups were analysed by one‐way ANOVA followed by Tukey's test. Data at different time points were compared using repeated‐measures ANOVA with Bonferroni's test

Subsequently, the impact of ASC‐EVs on HMECs was further investigated. After HMECs were transfected with miR‐486‐5p mimic, miR‐486‐5p inhibitor or ASC‐EVs alone or in combination, the cell migration rate was detected. Our results showed that compared with the control, increased migration rate was detected in the HMECs treated with ASC‐EVs and the HMECs transfected with miR‐486‐5p mimic. However, the treatment of ASC transfected with miR‐486‐5p inhibitor induced a decline in the migration rate of HMECs compared with the treatment of ASC‐EVs (Figure 4A‐D). Additionally, investigation of the proliferation of HMECs revealed enhanced cell vitality in HMECs treated with ASC‐EVs as well as miR‐486‐5p mimic relative to the control. However, the cells in HMECs treated in combination with the ASC‐EVs and miR‐486‐5p inhibitor were less active than the cells under the treatment of ASC‐EVs (Figure 4E).

The Matrigel‐based angiogenic assay was further performed to detect the effect of ASC‐EVs on HMECs. In contrast to the control, the treatment of ASC‐EVs, as well as the treatment of miR‐486‐5p mimic, led to the increased tube length and branch points. Whereas the tube length and branch points were decreased after the concomitant treatment of ASC‐EVs and miR‐486‐5p inhibitor (Figure 4F,G). Collectively, the above‐stated results suggested that ASC‐EVs‐secreted miR‐486‐5p promoted the proliferation and migration of HMECs as well as angiogenesis.

### 3.5 Sp5 is the direct target gene of miR‐486‐5p

**FIGURE 5:**
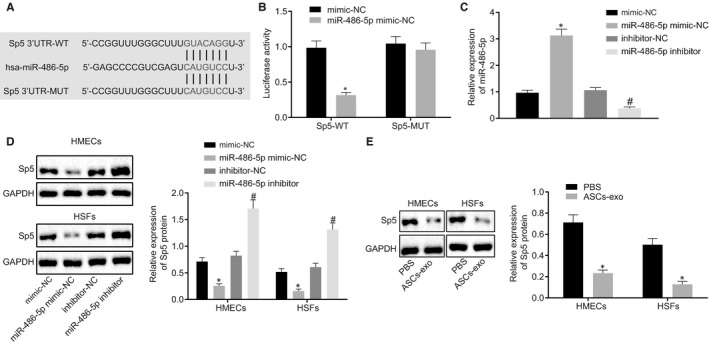
The targeting relation between miR‐486‐5p and Sp5. A, The specific binding sites between miR‐486‐5p and Sp5 predicted by an online website (http://www.targetscan.org/vert_71/). B, The luciferase activity in different treatment analysed by dual‐luciferase reporter gene assay. C, miR‐486‐5p expression in HMECs and HSFs with different transfection determined using RT‐qPCR. D, Sp5 protein level in cells with different treatments measured using Western blot analysis. E, Sp5 protein level in cells with or without ASC‐EVs studied using Western blot analysis. *P < 0.05 compared with the treatment of mimic NC or PBS. ^#^ *P* < 0.05 compared with inhibitor NC treatment. The measurement data were described as means ± standard deviation. Data between the two groups were analysed by unpaired *t* test

In an attempt to explore the downstream of miR‐486‐5p, the specific binding sites between miR‐486‐5p and Sp5 were predicted using the online database Targetscan, which displayed the binding sites between Sp5 mRNA and miR‐486‐5p at the location of 402‐408 (Figure 5A). The targeting relation was verified using the dual‐luciferase reporter gene assay. The luminescent signalling was reduced in the co‐transfection of miR‐486‐5p mimic with Sp5 3′UTR‐WT compared with the co‐transfection of mimic NC and Sp5 3′UTR‐WT. Whilst the luminescent signalling showed no significant difference between the co‐transfection of mimic NC with Sp5 3′UTR‐MUT and the co‐transfection of miR‐486‐5p mimic with Sp5 3′UTR‐MUT (Figure 5B). Besides, the miR‐486‐5p expression in HMECs and HSFs transfected with miR‐486‐5p mimic or miR‐486‐5p inhibitor was further evaluated using RT‐qPCR which demonstrated that the expression of miR‐486‐5p was up‐regulated whereas the Western blotting images and quantitative data of the protein bands revealed that Sp5 protein was decreased in cells transfected with miR‐486‐5p mimic. Furthermore, the treatment of miR‐486‐5p inhibitor resulted in a down‐regulated miR‐486‐5p expression and elevated Sp5 protein level (Figure 5C,D). Meanwhile, the Sp5 protein level was further verified by the Western blot analysis after HMECs and HSFs were treated with ASC‐EVs. Our results showed that the Sp5 protein expression in the treatment of ASC‐EVs was significantly declined than that in the treatment of PBS (Figure 5E). Taken together, the above‐reported findings revealed that miR‐486‐5p negatively regulates the expression of the target gene Sp5.

### 3.6 ASC‐EVs‐secreted miR‐486‐5p facilitates HSFs proliferation, migration and HMECs angiogenesis by inhibiting the expression of Sp5

**FIGURE 6:**
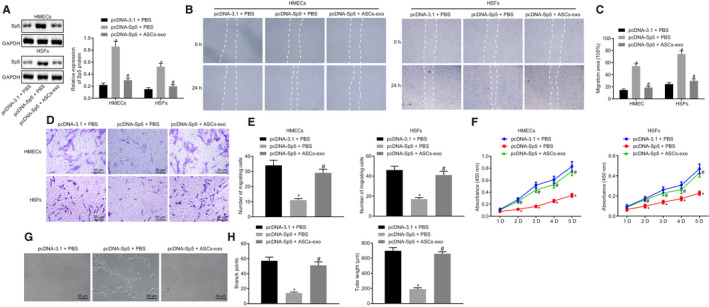
Angiogenesis of HMECs is promoted by ASC‐EVs‐secreted miR‐486‐5p/Sp5. A, The protein expression of Sp5 in HSFs and HMECs treated with overexpressed Sp5, ASC‐EVs or PBS studied using Western blot analysis. B and C, Migration ability of HSFs and HMECs quantified using the scratch test. D and E, Migration of HSFs and HMECs quantified using Transwell assay (×200). F, Proliferation of HSFs and HMECs examined by CCK‐8 assay. G and H, Angiogenesis of HMECs by quantifying the tube length and branch points in Matrigel‐based assay. (×200). *P < 0.05 compared with the treatment of pcDNA‐3.1 and PBS. ^#^ *P* < 0.05 compared with pcDNA‐Sp5 and PBS. The measurement data were described as means ± standard deviation. Data between two groups were analysed by unpaired *t* test, whilst data amongst multiple groups were analysed by one‐way ANOVA followed by Tukey's test. Data at different time points were compared using repeated‐measures ANOVA with Bonferroni's test

To further understand the mechanism through which HSFs and HMECs were affected by ASC‐EVs‐secreted miR‐486‐5p and its target gene Sp5, we overexpressed Sp5 in HSFs and HMECs which were then treated with ASC‐EVs or PBS. According to the Western blot analysis results, the protein expression of Sp5 was elevated in the treatment of pcDNA‐Sp5 and PBS; however, it declined after the treatment of pcDNA‐Sp5 and ASC‐EVs (Figure 6A). Scratch test and transwell assay unravelled that the cell migration rate in the treatment of pcDNA‐Sp5 and PBS was lower than that in pcDNA‐3.1 and PBS whilst the migration rate in the treatment of pcDNA‐Sp5 and ASC‐EVs was higher than that in the pcDNA‐Sp5 and PBS (Figure 6B‐E). Simultaneously, cell vitality, detected by CCK‐8 assay, was lowered in the treatment of pcDNA‐Sp5 and PBS but increased in the cells treated with pcDNA‐Sp5 and ASC‐EVs (Figure 6F). The above‐listed results indicated that ASC‐EVs delivered miR‐486‐5p facilitates the proliferation and migration of HSFs and HMECs by inhibiting the expression of Sp5.

Moreover, the angiogenesis amongst HMECs was evaluated by Matrigel‐based assay, which exhibited the reduced tube length and branch points in the treatment of pcDNA‐Sp5 and PBS whereas increased tube length and branch points were observed in the cells treated with pcDNA‐Sp5 and ASC‐EVs (Figure 6G,H). These findings collectively speculated that the ASC‐EVs‐secreted miR‐486‐5p was able to promote the angiogenesis by suppressing the Sp5 expression.

### 3.7 ASC‐EVs‐derived miR‐486‐5p boosts cutaneous wound healing

**FIGURE 7:**
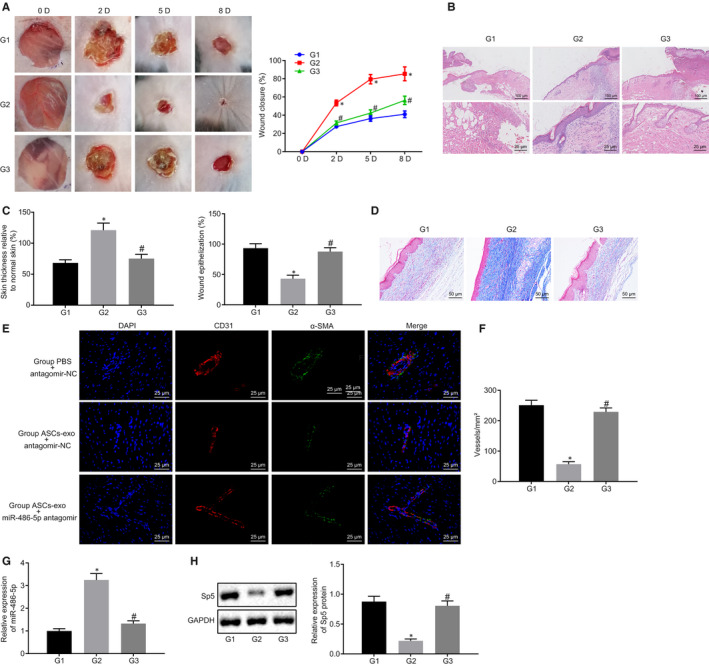
Wound area angiogenesis is promoted by ASC‐EVs delivered miR‐486‐5p. A, Would closure rate calculated in the wound treated with ASC‐EVs and/or miR‐486‐5p antagomir at the 2nd, 5th and 8th day after the surgical procedure. B, HE staining on the wound after 8 d post‐treatment with ASC‐EVs and/or miR‐486‐5p antagomir (up: 100 µm; down: ×400). C, Scar thickness and epithelialization quantification. D, Masson's trichrome staining on wounds treated with ASC‐EVs and/or miR‐486‐5p antagomir (×200). E, Immunofluorescence staining on CD31 in the wound treated with ASC‐EVs and/or miR‐486‐5p antagomir (×400). F, The extent of angiogenesis quantification on F. G, The expression of miR‐486‐5p in the wound tissues measured using RT‐qPCR (wounds treated with PBS and antagomir NC as the group 1; with ASC‐EVs and antagomir NC as the group 2; with ASC‐EVs and miR‐486‐5p antagomir as the group 3). H, The expression of Sp5 in the wound tissues measured using RT‐qPCR and Western blot analysis (wounds treated with PBS and antagomir NC as the group 1; with ASC‐EVs and antagomir NC as the group 2; with ASC‐EVs and miR‐486‐5p antagomir as the group 3). *P < 0.05 compared with the treatment of ASC‐EVs and antagomir NC. The measurement data were described as means ± standard deviation. Data between two groups were analysed by unpaired *t* test, whilst data amongst multiple groups were analysed by one‐way ANOVA followed by Tukey's test. Data at different time points were compared using repeated‐measures ANOVA with Bonferroni's test (with 5 mice in each group)

Aiming to study the ASC‐EVs delivered miR‐486‐5p impact on wound healing in mice, the skin wound was created on mouse dorsum with subcutaneous injection of ASC‐EVs or miR‐486‐5p antagomir. The wound was observed by photographing the wound, which displayed that the wound was healed more quickly after the wound was injected with ASC‐EVs and antagomir NC compared with the injection of PBS and antagomir NC. However, the injection of ASC‐EVs and miR‐486‐5p antagomir reduced wound healing. These findings were consistent with the results derived from the wound closure rating (Figure 7A). The results of HE staining suggested that more new skin, regenerative hair follicle, and adipocytes were found in the injection of ASC‐EVs and antagomir NC relative to the injection of PBS and antagomir NC whereas less new skin, regenerative hair follicle, and adipocytes were observed in the injection of ASC‐EVs and miR‐486‐5p antagomir (Figure 7B). Quantification of re‐epithelialization and scar thickness further confirmed that ASC‐EVs injection promoted the wound healing process whilst the addition of miR‐486‐5p exhibited contrary results (Figure 7C). The collagen was observed using Masson's trichrome staining, which showed that more curved collagenous fibres were presented in the treatment of ASC‐EVs relative to the control. However, the addition of miR‐486‐5p antagomir led to less curved collagenous fibres (Figure 7D).

The angiogenesis of the wound area affected by the miR‐486‐5p derived from ASC‐EVs was further analysed using the immunofluorescence staining on CD31. Meanwhile, the expression of miR‐486‐5p and Sp5 in the wound tissues were eventually measured using RT‐qPCR and Western blot analysis, respectively. Moreover, the fluorescence intensity of CD31 was enhanced, the extent of angiogenesis was increased, miR‐486‐5p expression was up‐regulated, and Sp5 expression was down‐regulated in the treatment of ASC‐EVs and antagomir NC relative to the PBS and antagomir NC injection. However, compared with the treatment of ASC‐EVs and antagomir NC, in the injection of ASC‐EVs and miR‐486‐5p antagomir, the fluorescence intensity of CD31 was weakened, the extent of angiogenesis and the expression of miR‐486‐5p was decreased, whilst the Sp5 expression was elevated (Figure 7E‐H). The above‐mentioned results indicated that ASC‐EVs‐secreted miR‐486‐5p stimulated the angiogenesis in the wound in vivo*.*

### 3.8 ASC‐EVs‐derived miR‐486‐5p stimulates cutaneous wound healing and wound area angiogenesis *via* Sp5/CCND2

**FIGURE 8:**
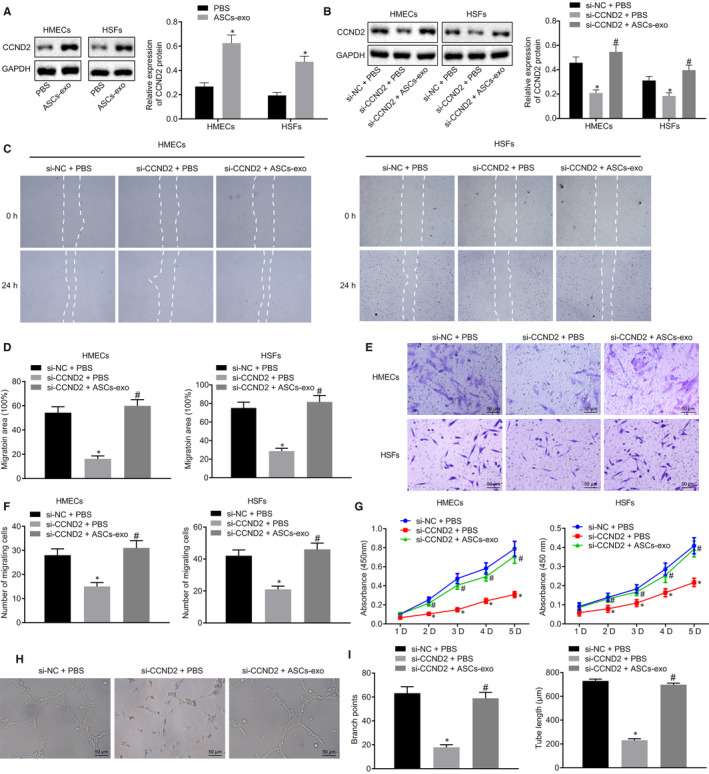
Proliferation and migration of HSFs and angiogenesis of HMECs are associated with ASC‐EVs‐derived miR‐486‐5p. A, CCND2 protein expression was detected by Western blot analysis after HSFs and HMECs were treated with ASC‐EVs. B, CCND2 protein expression in HSFs and HMECs was detected by Western blot analysis after treatment of siRNA against CCND2 and ASC‐EVs. C and D, The Migration ability of HSFs and HMECs quantified using the scratch test. E and F, Migration of HSFs and HMECs quantified using Transwell assay (×200). G, Proliferation of HSFs and HMECs examined by CCK‐8 assay. H and I, Angiogenesis of HMECs by quantifying the tube length and branch points in Matrigel‐based assay (×200). *P < 0.05 compared with the treatment of siRNA against NC and PBS. ^#^ *P* < 0.05 compared with siRNA against CCND2 and PBS treatment. The measurement data were described as means ± standard deviation. Data between two groups were analysed by unpaired *t* test, whilst data amongst multiple groups were analysed by one‐way ANOVA followed by Tukey's test. Data at different time points were compared using repeated‐measures ANOVA with Bonferroni's test

Lastly, to verify our assumption that ASC‐EVs‐secreted miR‐486‐5p could regulate the angiogenesis by binding to Sp5 and mediated the CCND2 expression, the expression of CCND2 in HSFs and HMECs was evaluated using Western blot analysis in the cells treated with ASC‐EVs. The protein level of CCND2 was distinctly increased in cells treated with ASC‐EVs relative to PBS (Figure 8A). The impact of CCND2 on the proliferation and migration of HSFs and HMECs was subsequently explored through the knockdown of CCND2 and the treatment of ASC‐EVs in cells successively. The transfection efficiency was detected by Western blot analysis, which showed that the protein level of CCND2 in the treatment of siRNA against CCND2 and PBS was lower than that in the siRNA against NC and PBS. In response to the treatment of siRNA against CCND2 and ASC‐EVs, the protein expression of CCND2 was enhanced (Figure 8B). Furthermore, the migration, vitality and angiogenesis of cells were examined using the scratch test, Transwell assay, CCK‐8 and Matrigel‐based assay. In the presence of siRNA against CCND2 and PBS, decreased cell migration, less active cells, reduced tube length and branch points were observed compared with the treatment of siRNA against NC and PBS. However, the delivery of siRNA against CCND2 and ASC‐EVs significantly enhanced cell migration, activate cells, augmented tube length and branch points (Figure 8C‐I). These results further validated that ASC‐EVs‐derived miR‐486‐5p potentially enhanced the proliferation and migration of HSFs as well as promote the angiogenesis of HMECs by regulating the Sp5/CCND2.

## 4 DISCUSSION

Cutaneous wound or injury accompanied by the damage of skin tissues amplify the risk for pathogens resulting in deleterious effects to the skin.^20^ Yet cutaneous wound healing remains a challenging process with multiple factors to be solved, particularly, angiogenesis, inflammation and oxidative properties.^21^ Besides, human ASCs are found in subcutaneous adipose tissues.^22^ Thus, amongst the established‐cutaneous wound healing strategies, adipose tissues are widely used for treatment options associated with the skin tissue regenerative medicine.^23^ In addition to its extensive application in wound healing, the positive impact of ASC‐derived EVs has also been discovered on angiogenesis or vascularization.^24^ Being potent mediators of inflammation and angiogenesis, the human ASC‐EVs‐secreted miRNAs are increasingly applied for the wound healing.^25^ In our study, we evidenced that miR‐486‐5p secreted from ASC‐EVs was able to boost cutaneous wound healing. Thus, in the present study, we provided evidence that ASC‐EVs‐derived miR‐486‐5p could potentially boost the process of cutaneous wound healing.

At the beginning of our study, we validated that ASC‐EVs‐secreted miR‐486‐5p literally promoted the angiogenesis and wound healing process. In the review of previous literature, miRNAs carried from EVs have been reported to accelerate the wound healing through the stimulation of fibroblast migration and proliferation.^26^ The aberrant expression of miR‐486‐5p, miR‐486‐3p, is addressed to promote cell proliferation in colorectal cancer cells.^27^ However, the ASC‐EVs miR‐486‐5p is rarely discussed before. The migration and proliferation of HSFs, detected by the scratch test, Transwell and CCK‐8 assay in our study, were promoted by the treatment of miR‐486‐5p secreted from ASC‐EVs, which further facilitated the HMECs angiogenesis. Fibroblasts are cells existed in the soft tissues, whose migration and proliferation are significant for collagen synthesis, tissue repair and wound healing.^28^ Consistent with our study, it has been reported that the specific miRNA, that is miR‐21 promoted the endothelial cell angiogenesis in myocardial infarction in vivo.^29^ Collectively these above‐described studies speculate that miR‐486‐5p secreted from ASC‐EVs hold the potential to enhance the wound healing process via promoting the migration and proliferation of HSFs and angiogenesis in HMECs.

Thereafter, we predicted and verified the Sp5 as the target gene of miR‐486‐5p. Sp5 has been largely investigated in the Wnt signalling. For instance, Sp5 acts as one of the inhibiting components in the Wnt3 signalling that is reported to induce angiogenesis and adipogenesis in preadipocytes.^30^, ^31^ However, scarce studies are focusing on the relationship between miRNAs and Sp5. Moreover, our data from the dual‐luciferase reporter gene assay demonstrated that ASC‐EVs‐secreted miR‐486‐5p negatively regulated the protein level of Sp5. To our best knowledge, we provided the evidence proposing the relation between miR‐486‐5p and Sp5.

Furthermore, we revealed that ASC‐EVs‐secreted miR‐486‐5p promoted the wound healing process by regulating the Sp5/CCND2. Nonetheless, Sp5, a transcription repressor has been demonstrated to inhibit the expression of CCND2.^13^ The overexpression of CCND2 has been attributed to the pro‐proliferation, boosting cell cycle and facilitating revascularization in human induced pluripotent stem cell‐derived cardiomyocytes.^32^ However, promoted CCND2 expression and inhibition of miR‐652‐3p expression are conductive to endothelial cell repair in atherosclerosis,^15^ which is partially consistent with our findings regarding the CCND2.

## 5 CONCLUSION

**FIGURE 9:**
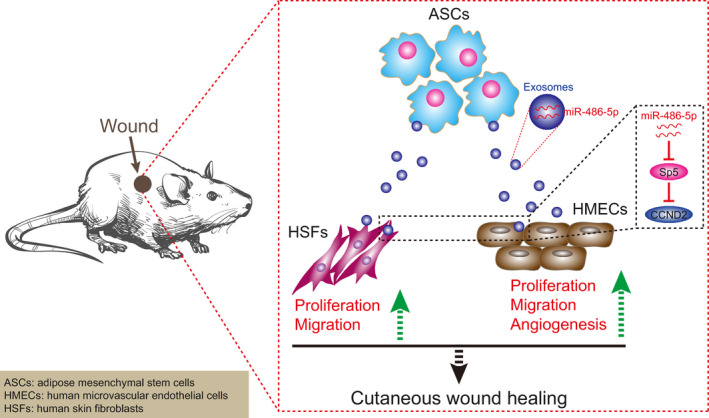
Mechanistic investigations show that ASC‐EVs‐derived miR‐486‐5p down‐regulates the expression of Sp5 and up‐regulates the CCND2 expression to promote the proliferation and migration of HSFs as well as the angiogenesis of HMECs thereby accelerating the cutaneous wound healing

In summary, the above‐discussed results demonstrated that miR‐486‐5p secreted from ASC‐EVs possesses the capacity to promote HSFs migration and proliferation as well as HMECs angiogenesis by inhibiting the expression of Sp5 and elevating the CCND2 expression (Figure 9). Thus, our data suggest a viable option for the stimulation of angiogenesis, which could have a potential application in cutaneous wound healing. For a comprehensive understanding of the therapeutic effect of ASC‐EVs‐secreted miR‐486‐5p, future studies are required to identify the optimum dose and the times of ASC‐EVs injection. The fact that previous studies have reported the role of several other miRNAs derived from ASC‐EVs on angiogenesis, however, our study remained focused on the mechanism of miR‐486‐5p due to the limitation of time and funding. Nonetheless, further investigations are prerequisites to explore the underlying mechanisms.

## CONFLICTS OF INTEREST

The authors declare no conflicts of interest.

## AUTHOR CONTRIBUTIONS

Yuanlin Zeng and Yingjie Lu designed the study. Huicai Wen and Jinjun Huang collated the data, carried out data analyses and produced the initial draft of the manuscript. Peng Liao; Huaiwei Liao and Jun Tu contributed to drafting the manuscript. All authors have read and approved the final submitted manuscript.

## Data Availability

The datasets generated/analysed during the current study are available.
